# Recombinant Expression and Functional Characterization of Martentoxin: A Selective Inhibitor for BK Channel (α + β4)

**DOI:** 10.3390/toxins6041419

**Published:** 2014-04-22

**Authors:** Jie Tao, Zhi Lei Zhou, Bin Wu, Jian Shi, Xiao Ming Chen, Yong Hua Ji

**Affiliations:** Lab of Neuropharmacology and Neurotoxicology, Shanghai University, Nanchen Road 333, Shanghai 200444, China; E-Mails: jietao@shu.edu.cn (J.T.); shupenguin@shu.edu.cn (Z.L.Z.); colabin@shu.edu.cn (B.W.); jshi@shu.edu.cn (J.S.); ming0911@shu.edu.cn (X.M.C)

**Keywords:** expression and purification, short-chain scorpion toxins, martentoxin, BK channels

## Abstract

Martentoxin (MarTX), a 37-residue peptide purified from the venom of East-Asian scorpion (*Buthus martensi* Karsch), was capable of blocking large-conductance Ca^2+^-activated K^+^ (BK) channels. Here, we report an effective expression and purification approach for this toxin. The cDNA encoding martentoxin was expressed by the prokaryotic expression system pGEX-4T-3 which was added an enterokinase cleavage site by PCR. The fusion protein (GST-rMarTX) was digested by enterokinase to release hetero-expressed toxin and further purified via reverse-phase HPLC. The molecular weight of the hetero-expressed rMarTX was 4059.06 Da, which is identical to that of the natural peptide isolated from scorpion venom. Functional characterization through whole-cell patch clamp showed that rMarTX selectively and potently inhibited the currents of neuronal BK channels (α + β4) (IC_50_ = 186 nM), partly inhibited mKv1.3, but hardly having any significant effect on hKv4.2 and hKv3.1a even at 10 μM. Successful expression of martentoxin lays basis for further studies of structure-function relationship underlying martentoxin or other potassium-channel specific blockers.

## 1. Introduction

The large-conductance, calcium-activated potassium channels (BK, also termed MaxiK) distributed in both excitable and non-excitable cells are involved in many cellular functions such as smooth muscle tone [1], neuronal firing [2], endocrine cell secretion [3], cell proliferation [4] and migration [5]. Functional BK channels are a tetramer of four pore-forming α subunits encoded by a single gene Slowpoke (Slo) [6]. Owing to the tissue-specific regulatory β-subunits and alternative splicing of Slo gene, BK channels possess a rather complex diversity of subtype family, which endow various physiological and pharmacological properties in different organisms [7,8,9].

Except for the long-chain sodium channel-specific modulators as valuable tools to exploring the obscure profiles of structure and function of their targets [10,11,12], fourteen short-chain K^+^-channel blockers (BmTX1-3, BmKTX, BmBKTX1, BmP01, BmP02, BmP03, BmP05, BmKK1-4, MarTX) have been isolated and characterized from the venom of *Buthus martensi* Karsch (BmK) [13,14,15,16,17,18]. A handful of them have been successfully hetero-expressed [1919].

**Figure 1:**
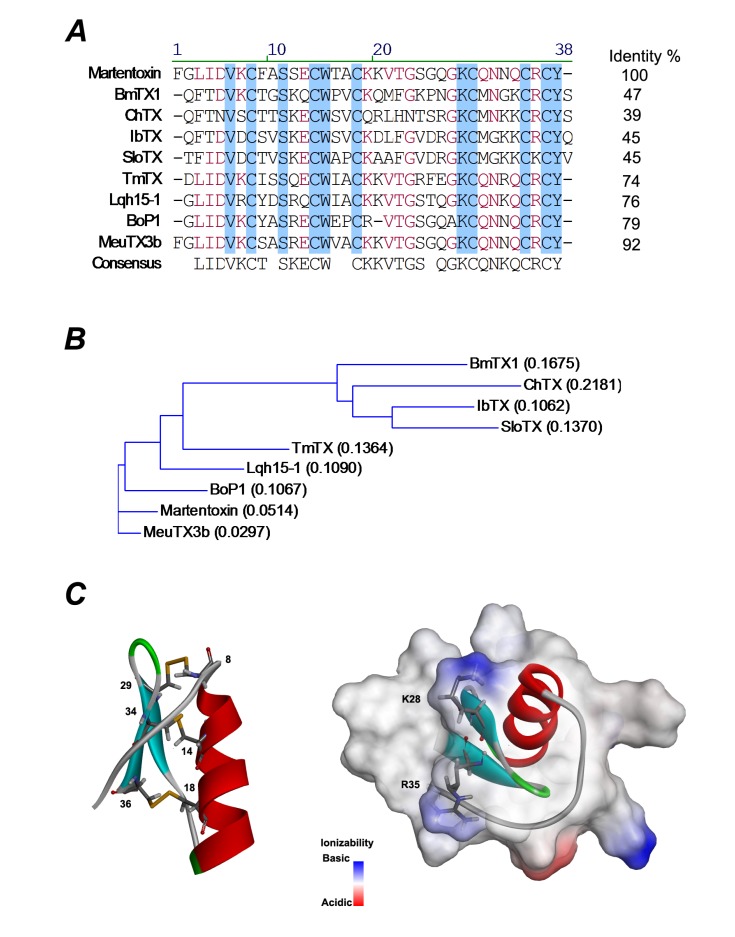
(**A**) Multiple sequence alignment of martentoxin and other K^+^ channel blockers from scorpion venom. Residues conserved among all these peptides are shadowed in blue; residues conserved in most of the peptides are in red. ChTX (charybdotoxin) and Lqh15-1 were purified from *Leiurus quinquestriatus* var. hebraeus; BmTX1 from *Buthus martensi* Karsch; IbTX (iberiotoxin) and TmTX (tamulotoxin) from *Buthus tamulus*; BoP1 from *Buthus occitanus* Paris; MeuTX3B from *Mesobuthus eupeus*; SloTX from *Centruroides noxius*; (**B**) A guide tree was constructed by ALIGNX, a component of the VECTOR NTI 8.0 software suite. Scores in the brackets are based on the identity of the amino acids’ chemical properties; (**C**) Representation of the mean structure of MarTX (PDB 1m2s) (left). Secondary structural elements are drawn with three disulfide bridges (yellow). The residues constituted to the disulfide bond are labeled. Molecular surfaces shown are solvent-excluded surfaces. The α-helix is in red and β-sheet strands are in light blue. The basic residues K28, R35 at β-face are highlighted.

Martentoxin, a 37-residue short-chain peptide belonging to α-KTx16 subfamily, is found to be specific to BK channels and intensively studied. By sequence comparison, martentoxin showed a poor sequential similarity (35%–50%) with those toxins in ChTX and KTX groups including BmTX1, BmTX2, BmTX3 and BmKTX from the same venom, but a high sequential identity (74%, 76%, 79% and 92%) with four other toxins, named as tamulotoxin (TmTX), Lqh15-1 [17], Bop 1 [20] and MeuTX3B [21] (Figure 1). Martentoxin consists of a triple-stranded antiparallel β-sheet (Figure 1C, blue) anchored to a single α-helix (Figure 1C, red) by three disulfide bridges (C8–C29, C14–C34, and C18–C36), which is shown in Figure 1C (left). Martentoxin could strongly block the currents of neuronal BK channels (α + β4) (IC_50_ = ~78 nM) [22]. In addition, when the concentration was increased to 10 μM, martentoxin only weakly inhibited the delayed rectifier potassium current (I_K_), and hardly affecting the transient outward potassium current (I_A_) in rat dissociated hippocampal neurons [23].

Due to the low amount of MarTX in BmK venom as well as the low yield efficiency by using conventional purification methods, an effective expression and purification approach was necessary to fully uncover the mode underlying the interaction of MarTX on BK channels. To this end, this study is committed to introducing the optimized-expression method for MarTX in pGEX-4T expression system. Finally, the pharmacological potency of recombinant MarTx was tested on three K^+^ channels.

## 2. Results and Discussion

### 2.1. Gene Expression and Purification of rMarTX

**Figure 2:**
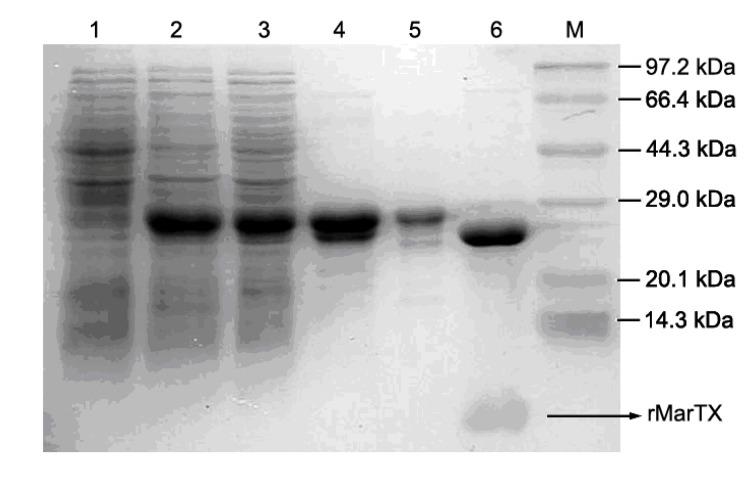
SDS-PAGE analysis. Lane M, protein molecular weight markers; lane 1, uninduced cell sample; lane 2, induced cell sample; lane 3, total soluble proteins extracted from the cytoplasm of induced cells; lane 4, purified fusion protein (GST-rMarTX); lane 5, desalted GST-rMarTX; lane 6, GST-rMarTX after enterokinase digestion (arrow indicates rMarTX).

The gel electrophoresis analysis of expressed and purified rMarTX is shown in Figure 2. Compared with the uninduced cell sample (lane 1), a new band appeared in the IPTG induced cell sample (lane 2). The estimated molecular weight of this new band was about 30 kDa, which was consistent with the theoretical molecular weight of the GST-rMarTX fusion protein. After sonication, the soluble cytoplasm extracts (lane 3) were subjected to chromatography column filled with Glutathione-Sepharose 4B for purification, which led to the partial purification of the 30 kDa product (lane 4). After a ultrafiltration procedure (10 kDa MWCO), the desalted fusion proteins were subjected to enterokinase digestion. The partially purified fusion protein (lane 5) was digested and released two major products (lane 6). The molecular weights of the resulting products were ~26 kDa and the band more less than 14 kDa, representing the GST tag and free rMarTX, respectively.

**Figure 3:**
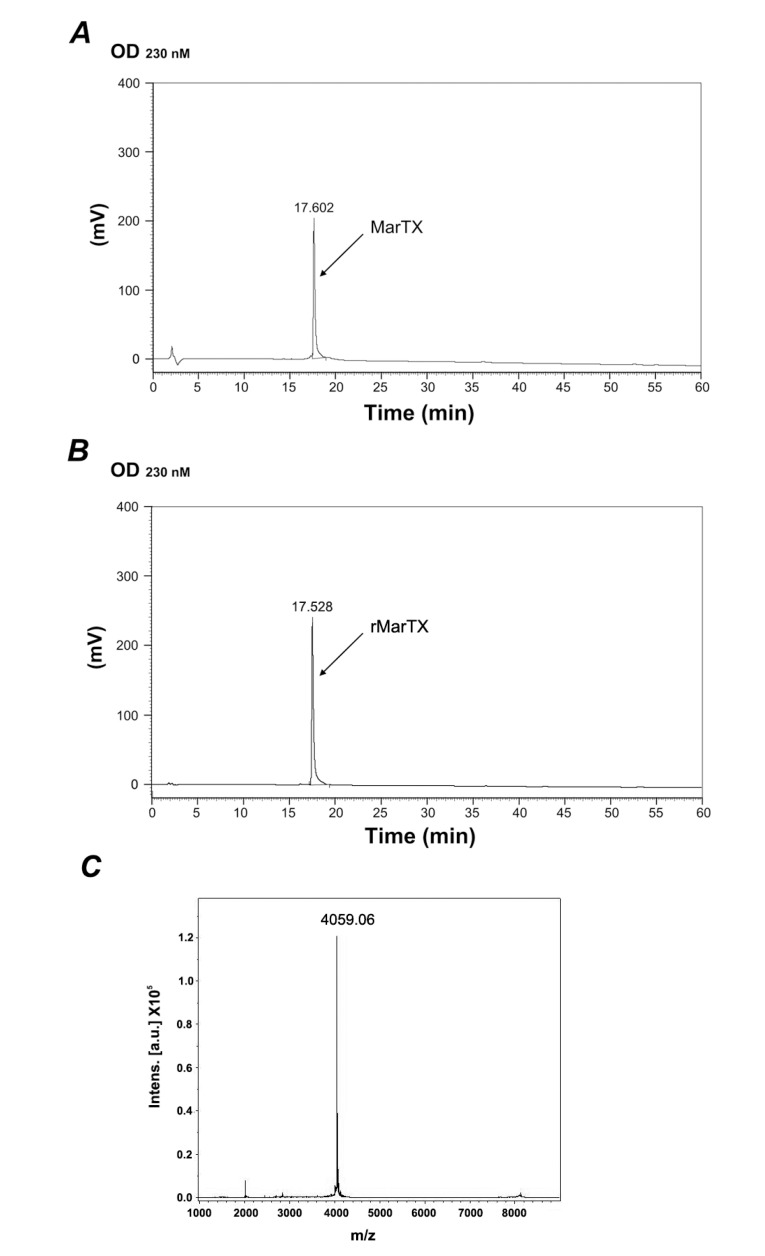
RP-HPLC chromatography and mass spectra of rMarTX. (**A**) RP-HPLC chromatography of native MarTX on a C18 column using a linear gradient of 5%–95% acetonitrile with 0.1% trifluoroacetic acid (TFA) in 60 min at a constant flow rate of 1 mL/min, and detected at 230 nm; (**B**) RP-HPLC chromatography of rMarTX under the same condition; (**C**) Mass spectra of purified rMarTX. The calculated theoretical molecular weight of rMarTX was 4060 Da [17] and the measured molecular weight was 4059.06 Da.

The enterokinase digested rMarTX was purified by RP-HPLC. A native MarTX sample was loaded into the C18 column as a control. The peak of control appeared at 17 min (Figure 3A). The peak corresponding to hetero-expressed MarTX also emerged at 17 min (Figure 3B). Eluted rMarTX of which the retention time close to control was collected, vacuum dried, and subjected to further analysis.

Figure 3C shows the mass spectrometry analysis results of the RP-HPLC purified product. The molecular mass was determined to be 4059.06 Da, less than 1-dalton difference compared with the theoretical molecular weight of native MarTX (4060 Da) [17]. The overall yield of recombinant toxin reached 0.61–1.31 mg from 1 L LB medium.

### 2.2. Inhibition of rMarTX on BK Channels (α + β4)

**Figure 4:**
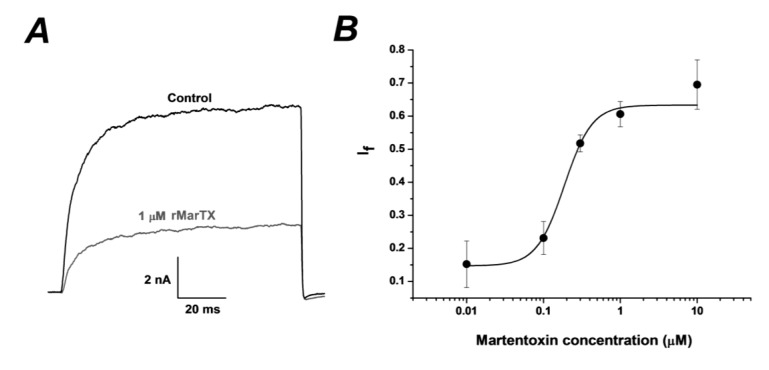
Inhibition of rMarTX on neuronal BK channels (α+β4) expressed in HEK293T cells. (**A**) Representative whole cell current traces from HEK 293T cells expressing BK channels (α+β4) before and after the application of recombinant martentoxin at 1 μM. The holding voltage was −70 mV and the currents were elicited by a pulse of +100 mV with 300 nM free Ca^2+^ in the pipette solution; (**B**) The dose-response curve of rMarTX inhibiting BK channel currents was fitted by the Hill equation (see ‘‘Data analysis’’). The IC_50_ value is 186.66 ± 0.04 nM, and the Hill coefficient is n = 2.41 ± 0.92 (n = 5–6).

To identify the pharmacological activity of rMarTX, BK channels (α + β4) was expressed in HEK293T cells. The currents evoked by BK channels (α + β4) are measured at +100 mV with typical characteristics as reported [9]. Hetero-expressed MarTX at 1 μM dose could potently inhibit the currents (Figure 4A), which was very close to the inhibitory effect of native MarTX at 1 μM [22]. The dose response curve was obtained and the percentage of block is shown as a function of rMarTX concentration (Figure 4B). The IC_50_ of rMarTX on BK channels was assessed to be ~186 nM, which was less than 2.5 times compared with the inhibitory effects of native MarTX (IC_50_ = ~78 nM). The The Hill coefficient is ~2.41, which was consistent with that of the native toxin [22].

### 2.3. Pharmacological Characterization of rMarTX on Kv Channels

**Figure 5:**
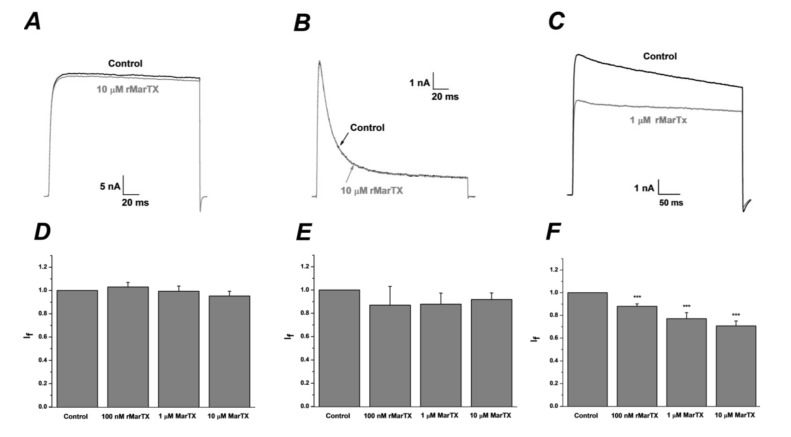
Effects of rMarTX on hKv3.1a, hKv4.2 and mKv1.3 channels. (**A**) Representative whole cell current traces from HEK 293T cells expressing Kv3.1a before and after the application of recombinant martentoxin at 10 μM. The holding voltage was −100 mV and the currents were elicited by a pulse of +40 mV; (**B**) Representative whole cell current traces from HEK 293T cells expressing Kv4.2 before and after the application of 10 μM rMarTX; (**C**) Representative whole cell current traces from HEK 293T cells expressing mKv1.3 before and after the application of recombinant martentoxin at 1 μM; (**D**) Statistics analysis of pharmacological modulation of Kv3.1 channels by 100 nM rMarTX (n = 9), 1 μM rMarTX (n = 9) and 10 μM rMarTX (n = 3) p > 0.05; (**E**) Statistics analysis of pharmacological modulation of Kv4.2 channels by 100 nM rMarTX (n = 5), 1 μM rMarTX (n = 5) and 10 μM rMarTX (n = 9) p > 0.05; (**F**) Statistics analysis of pharmacological modulation of mKv1.3 channels by 100 nM rMarTX (n = 10), 1 μM rMarTX (n = 8) and 10 μM rMarTX (n = 6) p < 0.001.

To identify the selectivity of rMarTX on other potassium channels, Kv 3.1 and Kv 4.2 were also expressed in HEK293T cells, respectively. The delayed rectifier potassium currents of hKv 3.1a channels evoked by +40 mV pulse could not be modulated by rMarTX even at 100 nM (I_f_ = 1.03 ± 0.04, n = 9, p > 0.05), 1 μM (I_f_ = 0.99 ± 0.04, n = 9, p > 0.05) or 10 μM (I_f_ = 0.95 ± 0.04, n = 3, p > 0.05, Figure 5A,D). Likewise, rMarTX has no significant effect on the transient outward potassium currents induced by hKv 4.2 channels at 100 nM (I_f_ = 0.87 ± 0.16, n = 5, p > 0.05), 1 μM (I_f_ = 0.88 ± 0.09, n = 5, p > 0.05) or 10 μM, respectively (I_f_ = 0.92 ± 0.06, n = 9, p > 0.05, Figure 5B,E). However, rMarTX could partly inhibit the currents of mKv1.3 channels at 100 nM (I_f_ = 0.88 ± 0.02, n = 10, p < 0.001), 1 μM (I_f_ = 0.77 ± 0.05, n = 8, p < 0.001) and 10 μM, respectively (I_f_ = 0.71 ± 0.04, n = 6, p < 0.001, Figure 5C,F).

### 2.4. Discussion

#### 2.4.1. Recombinant Expression of Martentoxin

Several successful cases of short-chain peptide expression in *E. coli* systems have been reported. The most widely employed strains are BL21 or its derivative BL21 (DE3). Some reports showed that the recombinant toxins were easy to accumulate as inclusion bodies in these expression systems, which reduced the activity and yield of recombinant toxins [24]. In our research, rMarTX at 1 μM could strongly inhibit the BK channel currents. The IC50 and Hill coefficient was very close to the native toxin. These results suggested that the bioactivity of recombinant martentoxin was not affected by this system.

On the other hand, in order to get a higher yield of rMarTX, we try to replace pGEX-4T-3 by other hetero-expression vectors (pET32a and pGEX-KG). However, through a series of experiments, the new systems had to be abandoned at last due to the low yield (Vector: pET32a Yield: N/A, Vector: pGEX-KG Yield: 42 μg/1 L LB) of expressing rMarTX.

#### 2.4.2. rMarTX as a Specific Probe for Neuronal BK Channels

Some potassium channel ligands such as iberiotoxin and charybdotoxin have been produced in *E. coli* with the bioactivities similar to natural toxins. In this study, the recombinant MarTx was also successfully expressed by the prokaryotic expression system, which exhibited higher affinity for neuronal BK channels (α + β4) rather than hKv4.2 and hKv3.1a channels. Moreover, due to the low inhibitiory-rate (I_f_ = ~0.71, Figure 5F) of rMarTX on mKv1.3 channels at high concentration (10 μM), rMarTx show higher selectivity for BK channels (α + β4). So far, as the classic blockers for BK channels, IbTX and ChTX have been widely used for investigating the function of BK channel subtypes. But even so, short-chain peptides are still rarely hetero-expressed as well as put into application. Accordingly, rMarTX has the great potential to be a specific tool for probing and fill the vacancies in finding specific blockers for neuronal BK channel (α + β4).

#### 2.4.3. Importance of N-Terminal Residue (Phe1) on Recognizing the BK Channels

It was reported that several peptide toxins, including dendrotoxin K, HmK (sea anemone Heteractis magnifica) and huwentoxin-I, were functionally expressed by pGEX system. Digested GST fusion proteins with thrombin, two additional amino acids were generated at N-terminal of these toxins. It was proved that the extra amino acids have no effect on biological activity of these toxins [25,26,27].

The structure of martentoxin is quite different from other toxins. From solution structure information and dynamic simulation results, the functional surface (β-face) of the molecule is characterized by less basic residues and extra aromatic residues. Among them, the N-terminal residue (Phe1) combined with the residue Phe266 of BK channel is essential for the formation of toxin-channel complexes [28]. Lqh15-1, sharing high-sequence identity with martentoxin, only were found to lack a Phe1 in the N terminus, which seemed to be ineffective on BK channels even at applied doses up to 300 nM [29]. In patch clamp recording, rMarTX including two additional amino acids at N-terminal, which was digested by thrombin, show low bioactivity. 100 μM of this rMarTX only inhibit 38% current amplitude of the BK channel in adrenal chromaffin cells (I_f_ = 0.38 ± 0.04, n = 4, [30]). In this study, two extra amino acids were removed by adding enterokinase cleavage sites, which result in facilitating the blockade of toxin on BK channels (α + β4).

#### 2.4.4. Pharmacological Significance of Martentoxin Acting on the Neuronal BK Channels

Epilepsies are disorders of neuronal excitability characterized by spontaneous and recurrent seizures. BK channels are critical for regulating neuronal excitability and contributing significantly to epilepsy pathophysiology. It is generally assumed that outward K^+^ currents through BK channels repolarize membrane and reduce cell excitability. However, in some neurons, the sharpening of action potentials due to increased BK channel activation (gain-of-function) has been found to facilitate high frequency firing [2]. As a specific neuronal BK channel inhibitor, martentoxin is possible to be selected as a therapeutic agent to reduce the effects of BK channel (gain-of-function) in facilitating abnormal activity and potentially, seizure initiation. Anticonvulsant action of martentoxin might be more effective by molecular modification in order to cross the blood-brain barrier. It may allow us to speculate that martentoxin could be utilized as a scaffold for designing novel molecules to alleviate symptoms of epilepsy.

## 3. Experimental Section

### 3.1. Materials

The *E. coli* strain DH5α used for plasmid cloning was purchased from TIANGEN (China). The expression vector pGEX-4T-3 and the *E. coli* host strain BL21 (DE3) were purchased from Novagen (USA). Enterokinase was purchased from New England Biolabs (USA). All restriction enzymes and other enzymes used in molecular cloning experiments were purchased from Takara Biotech (Japan) if not otherwise indicated. All chemicals and reagents were purchased from Sigma (USA). The synthesis of primers and the DNA sequencing of the constructed plasmids were performed by Lifetechnologies (China). The cDNA of martentoxin was kept in our laboratory [17].

### 3.2. Construction of pGEX-4T-3-Martentoxin Plasmid

**Figure 6:**
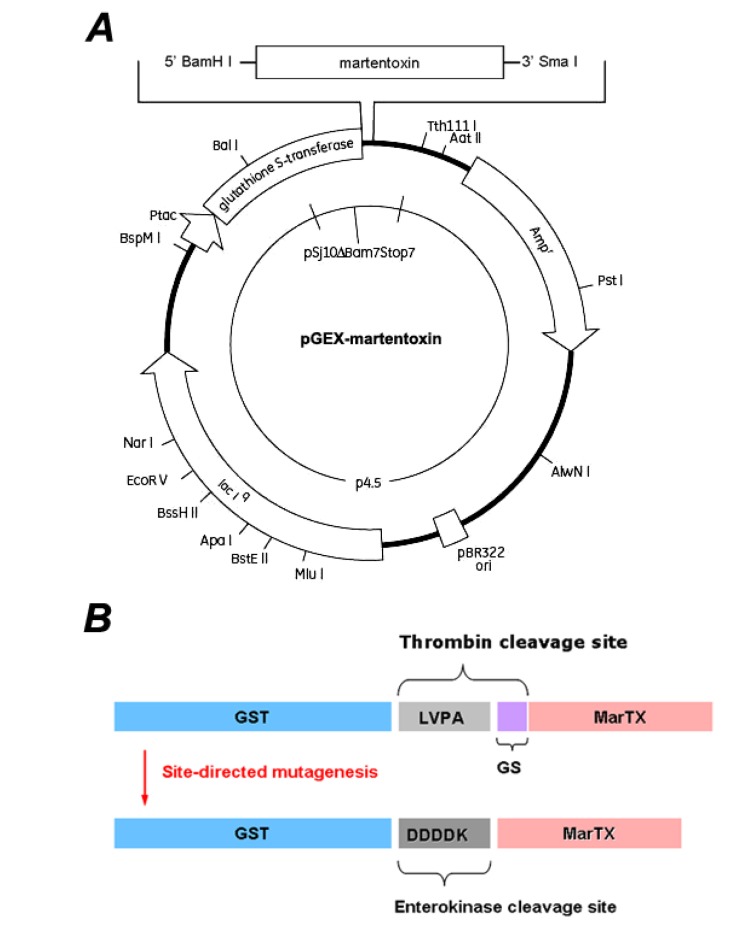
Construction of the pGEX-4T-3-rMarTX expression vector. (**A**) The gene martentoxin was cloned by PCR and fused via BamH I/Sma I sites; (**B**) The original expression vector, pGEX-4T-3-rMarTX including thrombin cleavage site (top). The mutant expression vector, pGEX-4T-3-rMarTX including enterokinase site (bottom).

Based on the cDNA sequence of MarTX (GenBank Accession No. AF534113.1) [17], two primers were designed to amplify the coding sequence of MarTX. The MarTX-sense primer (5’-TTCGGATCCTTTGGACTCATAGA-3’) contains a BamH I restriction site (underlined). The MarTX-anti-sense primer (5’-CTTCCCGGGTTAATCAGTAGCAT-3’) contains a Sma I restriction site (underlined). The cDNA of MarTX was inserted between the BamH I and Sma I sites present in pGEX-4T-3 in order to obtain the plasmid pGEX-4T-3-MarTX (Figure 6A).

In the plasmid pGEX-4T-3-MarTX, 6 extra bases were present between the thrombin cleavage site and the MarTX gene, resulting in 2 extra amino acid residues before the N-terminal of MarTX if expressed. Following a site-directed mutagenesis procedure described as the protocol of KOD mutagenesis kit (Toyobo, Japan), the additional bases were eliminated (Figure 6B). The sense primer (5’-GATGACGATGACAAGTTTGGACTCATAGACGTAAAATGTTTTG-3’) for PCR site-directed mutagenesis contains a DNA sequence of enterokinase cleavage site (underlined), whereas the anti-sense primer (5’-GGATCCACGCGGAACCAGATC) was the DNA sequence of thrombin cleavage site including a BamH I restriction site (underlined). The DNA sequences of all constructed plasmids were confirmed by DNA sequencing (Lifetechnologies, Shanghai, China).

### 3.3. Expression and Purification of rMarTX

The expression plasmid pGEX-4T-3-MarTX was transformed into *E. coli* BL21 (DE3) cells. *E. coli* cells were grown in 1L Luria-Bertani (LB) medium containing 0.1 mg/mL ampicillin at 37 °C until the cells reached a turbidity value of 0.4–0.6 at OD_600_. Isopropyl-β-d-thiogalactoside (IPTG) was added at a final concentration of 0.5 mM to induce toxin expression. Cells continued to grow at 28 °C for 4 h. After incubation, cells were harvested by centrifugation at 5000 g for 10 min at 4 °C and resuspended in 1 × phosphate buffered saline (PBS, 137 mM NaCl, 4.3 mM Na_2_HPO_4_, 2.7 mM KCl, 1.4 mM KH_2_PO_4_, pH 7.4), and sonicated on ice for 20 min (four bursts/min). The lysate was centrifuged at 12,000 g for 15 min at 4 °C. The supernatant was affinity purified with Glutathione-Sepharose 4B beads in Econo-Colum chromatography column (Bio-Rad Laboratories, Hercules, CA, USA) on ECONO PUMP (Bio-Rad Laboratories, Hercules, CA, USA) at the flow rate of 0.03 mL/min, washed with 30 mL ice-cold 1 × PBS buffer at the flow rate of 0.3 mL/min. GST-rMarTX fusion protein was eluted from beads by 10 mM Glutathione Elute Buffer (GEB) (Sigma-Aldrich, St. Louis, MO, USA). rMarTX was desalted by ultrafiltration tube (10 kDa MWCO, Millipore Corporation, Billerica, MA, USA) and cleaved from GST-rMarTX fusion protein by adding enterokinase at a concentration of 10 U/mL and incubated for 20 h at room temperature, The elution was subsequently purified by Sephadex G-50 column (General Electric, Fairfield, CT, USA) to remove GST tag at the flow rate of 15 mL/h. The elution peaks were detected by UV spectrophotometer at 215 nm wavelength. The purification was performed by repeated reverse-phase HPLC (Waters 600E-2487, Waters Milford, MA, USA) on a C18 column (Agilent Eclipes XDB-C 18, 4.6 mm × 150 mm, Agilent, Santa Clara, CA, USA) using a linear gradient of 5%–95% acetonitrile with 0.1% trifluoroacetic acid (TFA) in 60 min at a constant flow rate of 1 mL/min, and detected at 230 nm. The purified product was vacuum dried and reduced using 50 mM DTT in 0.05 M Tris-base buffer, pH = 8.0. The recombinant product was allowed to fold under controlled conditions using 2 M GndHCl in 0.05 M Tris-base buffer, pH 8.0, containing 1 mM reduced glutathione (GSH) and 0.1 mM oxidized glutathione (GSSG). The HPLC system was also used for the separation of the correct folded. All the washed and eluted proteins were analyzed by 15% SDS-PAGE. The correctly folded products were determined by MALDI-TOF mass spectra (Ultraflex™, Bruker Daltonics, Billerica, MA, USA). The yield of rMarTX was detected by an analytical balance (Mettler-Toledo, Greifensee, Switzerland).

### 3.4. Cell Culture and Transfection

The functional characterization of rMarTX was performed on the HEK 293T cells expressed with four kinds of K^+^ channels respectively as mentioned below. HEK 293T cells were obtained from Shanghai cell bank of Chinese Academy of Science. The cells were cultured in Dulbecco’s modified Eagle medium (DMEM; Life Technologies, Grand Island, NY) supplemented with 10% heat-inactivated fetal bovine serum (FBS; Gibco, Grand Island, NY). Culture dishes were incubated at 37 °C in a humidified atmosphere containing 5% CO_2_, and subcultured approximately every 2–3 days. The plasmids containing hSloα (U23767), β4 subunit (KCNMB4, AF207992), are kindly gifts from Noel Davies (University of Leicester), Jonathan Lippiat (Leeds university). Plasmids carrying hKv4.2 (KCND2, AJ010969.1) and hKv3.1a (KCNC1, NM_001112741.1) genes are donated by Ping Song (Yale University). The cDNA-encoding mKv1.3 was generously provided by George Chandy (University of California) and Ying Liang Wu (Wuhan University). All of the K^+^ channels mentioned above were transformed into HEK293T cells. One day before transfection, HEK 293T cells were transferred to 24-well plates. At 90% confluence, cells were transiently transfected using Lipofectamine2000 (Invitrogen, Carlsbad, CA, USA) at a ratio of 2 µL reagent with 1 µg total plasmid per well. Electrophysiological experiments were performed at 1–2 days after transfection.

### 3.5. Electrophysiological Recordings

Whole-cell voltage-clamp experiments were performed as described previously [22], using an EPC-9 amplifier (HEKA Eletronik, Lambrecht/Pfalz, Germany) at room temperature (21–25 °C). Patch pipettes were fabricated from glass capillary tubes by PC-10 Puller (Narishige, Setagaya-ku, Tokyo, Japan) with the resistance of 2–3 MΩ. Data acquisition and stimulation protocols were controlled by a Pentium III computer (Legend, Beijing, China) equipped with Pulse/PusleFit 8.3 software (HEKA Eletronik, Lambrecht/Pfalz, Germany). Capacitance transients were cancelled. Cells with a seal resistance (Rseal) below 1 GΩ were omitted. Series resistance (Rs) was compensated (80%–85%) to minimize voltage errors, and cells with an uncompensated series resistance (Rs) above 10 MΩ were omitted. Leak subtraction was performed using P/6 protocol. Data were low-passed at 10 kHz. The rate of solution exchange was studied using solutions with different KCl concentrations and found to be about 95% complete within 20 s. The holding potential was −70 mV for BK channels (α + β4), −100 mV for mKv1.3, hKv3.1a and hKv4.2 channels. The recordings were done with the pulse of +100 mV for BK channels (α + β4), +40 mV for mKv1.3, hKv3.1a and hKv4.2 channels.

### 3.6. Solutions

In the patch-clamp recordings, the standard bath solution for BK channels (α + β4) consisted of the following components (in mM): NaCl 135, KCl 5, MgCl_2_ 1.2, CdCl_2_ 2.5, HEPES 5, glucose 10 (pH 7.4 titrated with NaOH). Pipette solutions were composed of the following components (in mM): NaCl 10, KCl 117, MgSO_4_ 2, HEPES 10, MgATP 2, EGTA 1 (pH 7.2 titrated with KOH). The total Ca^2+^ to be added to give the desired free concentration was calculated using the program Maxchelator [31]. The bath solution for mKv1.3 and hKv3.1a contained the following (in mM): NaCl 135, KCl 5, MgCl_2_ 1, CaCl_2_ 1.8, HEPES 10, glucose 10 (pH 7.4 titrated with NaOH). The bath solution for hKv4.2 contained the following (in mM): NaCl 125, KCl 2, MgCl_2_ 1, glucose 10, HEPES 10, and TEA 20 (pH 7.4 titrated with NaOH). The pipette solution for mKv1.3, hKv3.1a and hKv4.2 contained the following (in mM): KCl 130, MgCl_2_ 0.5, MgATP 2, EGTA 10, HEPES 10 (pH 7.3 titrated with KOH). Unless otherwise stated, all reagents were purchased from Sigma.

### 3.7. Multiple Sequence Alignment and 3D Modeling

The multiple sequence alignment and guide tree are constructed by ALIGNX, a component of the VECTOR NTI 8.0 software suite (Lifetechnologies, Shanghai, China). 3D modeling and solvent-excluded surfaces of MarTX are constructed by Discovery Studio 4.0 (Accelrys,San Diego, CA, USA).

### 3.8. Data Analysis

Data were analyzed by PulseFit 8.5 (HEKA Eletronik, Lambrecht/Pfalz, Germany) and Origin 8.5 (Northampton, MA, USA). Results of data analysis were expressed as mean ± S.E.M. and n represents the number of the cells examined.

The statistical significance was determined using the unpaired Student’s t-Test or one-way ANOVA, and an asterisk denotes P < 0.05 unless otherwise stated. The degree of toxin effect was calculated by expressing the remaining current after each drug exposure as a fraction of the current magnitude of the patch prior to the first drug exposure (*i.e*., fractional current remaining, I_f_).

Dose-response curve for the percent enhancement of BK channel (α + β4) currents was drawn according to the Hill equation I = Im/(1 + ([toxin]/IC_50_)^n^), where Im is maximum enhanced percentage of BK currents, and [toxin] is the concentration of martentoxin. IC_50_ (half-maximal inhibitory concentration) and n denote the toxin concentration of half-maximal effect and the Hill coefficient, respectively.

## 4. Conclusions

The short-chain scorpion peptide (martentoxin) has been hetero-expressed by the modified pGEX-4T-3 expression system, in which the thrombin cleavage site was replaced by enterokinase site. Two extra amino acids at the N-terminal of recombinant peptide were successfully removed through this vector transformation. The molecular weight and bioactivity of the recombinant peptide (martentoxin) was almost consistent with the natural polypeptide. The recombinant peptide (martentoxin) could selectively inhibit the currents of BK channels (α + β4), partly inhibit mKv1.3, but hardly had any significant effect on Kv4.2 and Kv3.1a channels.
